# Evaluation of Antigenic Comparisons Among BVDV Isolates as it Relates to Humoral and Cell Mediated Responses

**DOI:** 10.3389/fvets.2021.685114

**Published:** 2021-06-15

**Authors:** Shollie M. Falkenberg, Rohana P. Dassanayake, Brett Terhaar, Julia F. Ridpath, John D. Neill, James A. Roth

**Affiliations:** ^1^Ruminant Disease and Immunology Research Unit, National Animal Disease Center, United States Department of Agriculture (USDA), Agricultural Research Service, Ames, IA, United States; ^2^Frontier Veterinary Research and Consulting, Winterset, IA, United States; ^3^Ridpath Consulting, LLC, Gilbert, IA, United States; ^4^Department of Veterinary Microbiology and Preventive Medicine, College of Veterinary Medicine, Iowa State University, Ames, IA, United States

**Keywords:** antigenicity, antigenic diversity, bovine viral diarrhea virus, virus neutralizing titer, cell mediated response

## Abstract

Antigenic differences between bovine viral diarrhea virus (BVDV) vaccine strains and field isolates can lead to reduced vaccine efficacy. Historically, antigenic differences among BVDV strains were evaluated using techniques based on polyclonal and monoclonal antibody activity. The most common method for antigenic comparison among BVDV isolates is determination of virus neutralization titer (VNT). BVDV antigenic comparisons using VNT only account for the humoral component of the adaptive immune response, and not cell mediated immunity (CMI) giving an incomplete picture of protective responses. Currently, little data is available regarding potential antigenic differences between BVDV vaccine strains and field isolates as measured by CMI responses. The goal of the current paper is to evaluate two groups of cattle that differed in the frequency they were vaccinated, to determine if similar trends in CMI responses exist within each respective group when stimulated with antigenically different BVDV strains. Data from the current study demonstrated variability in the CMI response is associated with the viral strain used for stimulation. Variability in IFN-γ mRNA expression was most pronounced in the CD4^+^ population, this was observed between the viruses within each respective BVDV subgenotype in the Group 1 calves. The increase in frequency of CD25^+^ cells and IFN-γ mRNA expression in the CD8^+^ and CD335^+^ populations were not as variable between BVDV strains used for stimulation in the Group 1 calves. Additionally, an inverse relationship between VNT and IFN-γ mRNA expression was observed, as the lowest VNT and highest IFN-γ mRNA expression was observed and vice versa, the highest VNT and lowest IFN-γ mRNA expression was observed. A similar trend regardless of vaccination status was observed between the two groups of calves, as the BVDV-1b strain had lower IFN-γ mRNA expression. Collectively, data from the current study and previous data support, conferring protection against BVDV as a method for control of BVDV in cattle populations is still a complex issue and requires a multifactorial approach to understand factors associated with vaccine efficacy or conversely vaccine failure. Although, there does appear to be an antigenic component associated with CMI responses as well as with humoral responses as determined by VNT.

## Introduction

Successful bovine viral diarrhea virus (BVDV) control strategies generally involve a multipronged approach that incorporates detection (testing/culling), intervention measures (vaccination), and biosecurity (1). All approaches and implementation strategies should be considered to determine the most appropriate and cost-effective approach for each individual farm or entity as it relates to control programs. The current paper will focus on vaccination as a method of control and antigenic differences between vaccine and field strains as a potential gap associated with reduced protection.

Understanding the reason for reduced vaccine protection is critical to the design of effective control programs. Because vaccination is a relatively inexpensive and effective method of control and it is often the first and frequently only method used in regions with a limited BVDV control program. Further, the highest BVDV prevalences were observed in countries that failed to implement any intervention strategy such as vaccination (2). The goal of vaccination, as a method for controlling BVDV infections, is to reduce or prevent viremia in animals which subsequently may lead to prevention of fetal infections (3, 4). Preventing viremia is critical for reducing transmission/shedding of the virus within a population of animals and thus reducing the impact of infection.

BVDV vaccines have been available since the 1960's and studies conducted under controlled conditions have shown vaccines to be efficacious in reducing disease and transmission (5). While vaccination has been demonstrated to be an effective method of control, vaccination as a stand-alone control measure has not resulted in a significant reduction in prevalence or losses associated with BVDV (4, 6). The limited control seen with vaccines does not mean that vaccines cannot be an effective control tool. However, it appears that shortfalls in control programs based solely on vaccination are associated with the heterogeneity of BVDV field strains, the ability of these viruses to establish persistent infections, and the greater level of protection needed to prevent fetal infections (3).

BVDV, belong to the *Pestivirus* genus and are divided into two species, namely BVDV-1 and BVDV-2 (classified as *Pestivirus A* and *B*, respectively) (7). Multiple regions of the BVDV genome have been explored for genetic characterization, and recent advancements in diagnostic methods, sequencing and phylogenic analysis have identified 21 BVDV-1 subgenotypes (BVDV-1a through 1u) and four BVDV-2 subgenotypes (BVDV-2a through 2d) (8). Genetic comparisons are useful for segregation of BVDV isolates into genotypes and determination of prevalence of those isolates within populations. However, there does not appear to be an established measurement or criterion that correlates the relationship between genetic and antigenic similarities and differences. The inability to accurately determine antigenic similarities or differences between isolates makes the development of broadly protective vaccines difficult. Rather than genetic comparisons, serology was initially evaluated for classification of BVDV isolates into serological subgenotypes. Given the heterogeneity among BVDV isolates, and cross-neutralization among isolates, serological subgroups were not recognized. Although, an important example of the impact of pestiviruses antigenic diversity is the addition of BVDV-2 strains in the composition of vaccines (9, 10). Currently, the most predominant subgenotype detected in BVDV PI calves in the US is BVDV-1b (11, 12), and this predominance is significant since no US licensed vaccines include BVDV-1b as a component. In addition, other genetically diverse BVDV-1 and−2 isolates belonging to 1c, 1i, 2b, and 2c subgenotypes that are not contained in any vaccine have also been identified in the US (13, 14). Considering the increased genetic diversity observed for BVDV isolates detected within the US and globally, a better understanding of the relationship between genotypes and antigenic divergence is critical as it relates to BVDV control strategies, specifically vaccines and the failure to protect.

There are a limited number of studies that have evaluated the serological relationships among BVDV subgenotypes (15–19). These studies highlight the antigenic variability not only between BVDV species but also between genetic subgenotypes. Although, no discernable antigenic differences or similarity patterns could be discerned when collectively evaluating the data from these studies. The antigenic similarities and differences observed in these studies appeared to be isolate and study specific rather than a general trend among all isolates that belong to a subgenotype. Most recently, a multivariate analysis for determining antigenic relatedness among Pestiviruses was described (20). Using this methodology, antigenic diversity was demonstrated not only among BVDV species, but also among BVDV-1 subgenotypes. Data from the multivariate analysis would suggest that some BVDV-1 strains are as antigenically distinct from each other as BVDV-2 strains are distinct from BVDV-1 strains (20). While other studies were unable to discern serological subgroups (15), the multivariate analysis appears to provide clusters of strains that have similar VNT patterns and may be a method for better understanding BVDV serologic subgroups.

Using serology to evaluate antigenic comparisons can be complex and difficult to interpret. Given the diversity of BVDV reference strains used in studies (8), lack of reference sera, and variations in methods used to determine VNT, it is difficult to make direct comparison among studies. Further, the practical significance of generalizations based on VNT can be problematic as information on level of protection required to prevent disease and/or fetal infection is limited. Previous studies have described a VNT ≥ 1:512 is required for marked protection (21) whereas a VNT of 1:256 was found to be critical for the prevention of clinical signs (22). In one study colostrum deprived calves were fed various amount of colostrum to establish a range in titer of passively acquired viral neutralizing antibody in the serum (22), whereas an inactivated BVDV vaccine was used to elicit viral neutralizing antibody in the other study (21). It is unknown if the approaches used to generate antibodies in each respective study could have impacted the conclusion of titer necessary for prevention of clinical disease. Furthermore, these studies are evaluating protection from clinical disease, rather than prevention of fetal infection, which may require greater protection. Previous studies utilizing currently available modified live viral (MLV) vaccines have demonstrated fetal protection against BVDV-1b strains, which are not currently included in licensed MLV vaccines (23, 24). Although, while fetal protection was conferred using a currently available MLV vaccine against BVDV-1b and 2a PI's, a BVDV-1a PI was detected in cows vaccinated with the MLV vaccine. Furthermore, it has been demonstrated that BVDV MLV vaccines can induce cell mediated responses (25–27), but recent fetal protection studies only report VNT and do not measure CMI (23, 28). Therefore, it is unknown if a potential reason associated with lack of protection, in apparently effectively vaccinated animals, could be associated with failure to induce CMI response to complement the humoral response. Therefore, to better understand the role CMI has in protection against genetically and serologically distinct strains, two groups of non-vaccinated and vaccinated cattle were utilized. The two groups of cattle were utilized to evaluate if similar trends in CMI responses were observed in cattle that differed in the frequency they were vaccinated. CMI responses have been previously reported and generally are characterized by the induction of IL-2, IFN-γ, and CD25 labeling in vaccinated calves as compared to non-vaccinates (25–27, 29, 30). Therefore, these measures were used in the current study to evaluate differences among different BVDV strains as measures of antigenic differences in different PBMC populations.

## Materials and Methods

### Animals and Sample Collection

Animals housed, and samples collected at the National Animal Disease Center were handled in accordance with the Animal Welfare Act Amendments (7 U.S. Code §2131 to §2156). All procedures were approved by the Institutional Animal Care and Use Committee of the National Animal Disease Center (ARS-2018-720).

A subset of data and whole blood samples referenced in this study from Group 2 calves were collected during procedures by a private party and analyzed for this publication. The samples were generated during processing by the private party and were submitted as diagnostic specimens resulting in no oversight by the Institutional Animal Care and Use Committee of the National Animal Disease Center.

Two groups of cattle (Group 1 and Group 2) were utilized to evaluated CMI responses. Group 1 consisted of five Holstein steers that tested negative for BVDV antigen and antibody and were previously utilized for validation of the cell mediated assay utilized in the current study (27) and were utilized as assay controls to screen different BVDV strains. These steers were used for screening purposes as previous it had been demonstrated they had significant responses associated with CMI (27). Briefly, three calves served as positive controls and were administered commercially available pentavalent MLV vaccines containing BVDV type 1 and type 2, bovine herpes virus-1 (BHV-1), bovine respiratory syncytial virus (BRSV), and bovine parainfluenza type 3 virus (PI-3). Each calf received exclusively one of the following commercially available vaccines; BoviShield Gold 5 (NADL_BVDV-1a and 53637_BVDV-2a), Titanium 5 (C24V_BVDV-1a and 296c_BVDV-2a), or Pyramid 5 (Singer_BVDV-1a and 5912_BVDV-2a). Two calves served as negative controls and were administered sterile PBS at the same volume and route as the vaccinated calves. Timing of re-vaccination and sampling are as previously described (27). Briefly calves were vaccinated at ~4–5 months of age and re-vaccinated every 3–4 months, receiving a total of 3 doses of vaccine. Beginning 12 weeks following the last dose of vaccine (3^rd^ vaccination; ~10–11 months after initial vaccination) two sequential sample collections were obtained over the course of a 2-week period. Blood samples were collected via jugular venipuncture in tubes containing acid citrate dextrose (BD Vacutainer ACD, Franklin Lakes, NJ) for isolation of PBMCs and in serum separation tubes with gel and clot activator (BD Vacutainer SST, Franklin Lakes, NJ) for serum.

Group 2 cattle belonged to a private party and consisted of commercial Charolais purebred cattle approximately 6 months of age at the time of vaccination. Eight calves were administered a commercially available pentavalent MLV vaccine (Titanium 5^®^) containing BVDV-1a (C24V) and BVDV-2a (296c), BHV-1, BRSV, and PI-3. Eight negative control calves did not receive a MLV vaccine and remained unvaccinated over the course of the study period. Approximately 12 weeks post vaccination, samples were collected to evaluate CMI responses against BVDV-1a, 1b, and 2a isolates.

### Virus

Non-cytopathic (ncp) field isolates, representing the predominant BVDV species and subgenotypes already described in US, were selected for this study based on the sequence diversity observed in the open reading frame (ORF). The ORF encodes a large polyprotein consisting of four structural (C, E^*rns*^, E1, E2) and eight non-structural proteins (N^*pro*^, p7, NS2, NS3, NS4A, NS4B, NS5A, and NS5B). The field isolates were also chosen based on the previously described antigenic diversity as determined by virus neutralization (VN) assay and principle component analysis (20). Isolates were selected to represent both the range of genetic diversity observed in the phylogenetic analysis and the range of serological antigenic diversity observed in the principle component analysis. Based on the afore mentioned analysis 12 field isolates representing the BVDV-1 (1a and 1b) and BVDV-2a subgenotypes. A total of four BVDV-1a (BOAEC1190, GL760, PI34, and PI407), four BVDV-1b (Nebraska, PI11, PI285, and PI819), and four BVDV-2a (890, MARC-60760, PI28, and AzSpleen) were selected. Details regarding complete genome sequencing and BVDV isolate characterization are previously described in the literature, in addition to GenBank accession numbers (20).

All viruses were propagated in Madin Darby bovine kidney (MDBK) cells that had been tested and free of BVDV and HoBi-like viruses as previously described (31). Cells were grown in complete cell culture medium composed of minimal essential media (MEM; Sigma-Aldrich, St. louis, MO), supplemented with L-glutamine (1.4 mM; Gibco, Life Technologies, Grand Island, NY), 1% of antibiotic-antimycotic-100× consisting of Streptomycin, Amphotericin B, and Penicillin (Invitrogen, Life Technologies, Carlsbad, CA), and 10% FBS(PAA, Ontario, Canada) that was heat inactivated. FBS was tested and found to be free of BVDV and HoBi-like antigens and antibodies. Culture flasks were freeze-thawed, and culture medium was centrifuged at 500×*g* for 10 min and passed through a 0.22-μm filter to remove any cell debris. Viral titers were determined via dilution on a primary BTu cell line derived from fetal bovine turbinate cells (32). Endpoints were determined based on immunoperoxidase staining using the monoclonal antibody N2 developed in our lab, which binds the E2 protein of the bovine pestiviruses used in this study and previously described (9, 33).

### Isolation, Culture, and Preparation of Peripheral Blood Mononuclear Cell (PBMC) for Flow Cytometry

Peripheral blood mononuclear cell (PBMC) isolation was conducted as previously reported (27), with the exception that a Muse^™^ Cell analyzer was used to determine the cell count and viability function, per the manufacturer recommendation, to standardize the total number of live PBMC cells present in each sample. Live cells are defined as total number of PBMCs that did not stain as dead cells with the manufacture's propriety viability stain (Muse^™^ Count and Viability Reagent; MilliporeSigma, Burlington, MA). Total live cells values were used to adjust samples for each calf so that all assays used the same number of cells (~ 1 × 10^6^). Adjusted cell suspensions were centrifuged at 300 × *g* for 5 min and the cell pellet was resuspended in complete RPMI-1640 supplemented with 10% (v/v) heat-inactivated FBS, and antibiotic-antimycotic as previously described. Two hundred μl of each PBMC suspension containing ~ 1 × 10^6^ cells were added to respective wells of a 96-well round bottom plate. Cells were plated in duplicate for each respective non-stimulation or stimulation method for each calf. Plated cells were incubated at 37°C in a humid atmosphere of 5% CO_2_ for the duration of the stimulation period. After 24 h, 50 μl of media was removed from the respective wells for each calf and replaced with 50 μl of each respective BVDV virus previously described in virus preparation at an approximate MOI of 1. Forty-eight hours after cells were plated, 50 μl of media was removed from the wells designated for mitogen stimulation and 50 μl of eBiosciences cell stimulation cocktail (PMA/ionomycin; 8 μl diluted in 1 mL complete RPMI-1640) was added. The mitogen stimulated cells were included as positive controls for the assay. Two remaining wells were not stimulated and were used as non-stimulated controls. Two hours after the addition of cell stimulation cocktail, all plated cells were prepared for use in the flow cytometry assay.

The list of mAb combination for identification of PBMC subpopulations, panel configuration and reagents used are summarized in detail (Table 1). Briefly, the primary mAbs used consisted of; mouse anti-bovine CD2 (Clone MUC2A, Isotype IgG2a), mouse anti-bovine CD8 (Clone BAQ111A; Isotype IgM), mouse anti-bovine CD25 (Clone LCTB2A; Isotype IgG3) and mouse anti-bovine CD335 (Clone AKS1; Isotype IgG1). All primary mAbs were purchased from Washington State University (WSU) Monoclonal Antibody Center (Pullman, WA) with the exception of CD335 (Bio-Rad, Hercules, CA). All mAbs were diluted at 1:100 dilution in stain buffer (BD Biosciences, San Jose, CA). Secondary Ab conjugates added wells containing the respective isotypes and consisted of; goat anti-mouse IgM-Brilliant Violet 711 (BD BioSciences, San Diego, CA), goat anti-mouse IgG1-PE/Cy7 (Southern Biotechnology Associates, Birmingham, AL), goat anti-mouse IgG2a-Brilliant Violet 421 (BioLegend, San Diego, CA), and goat anti-mouse IgG3-BUV395 (BD BioSciences). Flow cytometric analysis was performed using a BD FACSymphony^™^ A5 flow cytometer (BD BioSciences). Compensation beads from the PrimeFlow kit as we as CompBeads (BD BioSciences) were used to set up compensation for each fluorochrome. While positive signals were evident, single stain controls and fluorescence-minus-one controls were evaluated to optimize acquisition gates and compensation for each fluorochrome/channel. Cells were visualized in forward and side light scatter and electronic gates were placed on the scatter region that contained live cells. Doublet discrimination was then used to analyze single cells. At least 50,000 events were collected for each sample for data analysis.

**Table 1:** Primary and secondary antibodies used for surface marker expression on PBMC's and Primeflow probes used for cell mediated immune response comparisons.

Primary antibody^*^	**Cell marker**	**Clone**	**Isotype**	**Fluorochromes**
CD2	T and NK cells	MUC2A	IgG2a	BV421
CD8α	T cell subset	BAQ11A	IgM	BV711
CD25	IL-2 receptor/activation	LCTB2A	IgG3	BUV395
CD335	NK cells	ASK1	IgG1	PE/Cy7
CD4 PrimeFlow probe	T cell subset			AF568
IL-2 PrimeFlow probe	IL-2 mRNA/stimulation			AF750
IFN-γ PrimeFlow probe	IFN-γ mRNA/stimulation			AF488
BVDV PrimeFlow probe	BVDV viral RNA			AF647

* *CD, cluster of differentiation*.

The sequence of the genomic region coding for N^pro^-C-E^rns^ (~1,500 nt) of each respective BVDV strain used for stimulation was provided to Thermo-Fisher Scientific (Waltham, MA) to design gene-specific oligonucleotide (RNA) target probes for each BVDV strain. *Bos taurus-*specific probes for IFN-γ, IL-2, and CD4 were commercially available through the manufacturer.

At the end of the culture period, approximately 48 h post-isolation, 24 h post-BVDV stimulation, and 2 h post mitogen stimulation, cells were prepared for flow cytometry and analyzed as previously described (27). The mitogen-stimulated and non-stimulated PBMCs were included to; validate the functionality and optimize acquisition gates to detect the presence of IFN-γ and IL-2 in cultured PBMCs and control for background.

### Virus Neutralization Assay

VN assays were performed according to previously described protocol (22) using the serum collected from Group 1 calves approximately 12 weeks post-third vaccination (10–11 months post-initial vaccination) and ~12 weeks post-vaccination for Group 2 calves. Serial two-fold dilutions of each antiserum in MEM were prepared, starting from a 1:2 initial dilution. In cell culture 96-well microplates, using replicates of five wells for each serum dilution, a 50-μl aliquot of diluted serum and a 50-μl aliquot of virus containing 100 TCID50 were added to each well and incubated for 1 h at 37°C. At the end of the incubation period, a primary BTu cell line derived from fetal bovine turbinate cell was added. This was accomplished by addition of 20,000 BTu cells (in a 100-μl aliquot of DMEM and 10% FBS) to each well. Microplates were incubated for 4–5 days at 37°C in a 5% CO2 incubator. Replication of the virus was tested using monoclonal antibody N2 and horseradish peroxidase-conjugated protein G as previously described (34) for ncp isolates, whereas CPE was evaluated for cp strains. Wells without any observable CPE or cell layer staining in each serum dilution were used for the calculation of the endpoint through Spearman-Kärber method, as previously described (35).

### Data Analysis

The frequency of cells staining positive for the respective PBMC populations (CD2^+^, CD4^+^, CD8^+^, and CD335^+^) was calculated for each sample using FlowJo^®^ software (Tree Star, Inc.). Within each cell population evaluated, the frequency of positive cells for CD25, BVDV, IFN-γ, and IL-2 was determined. The frequency (percent positive) cells for IFN-γ and IL-2 was determined by subtracting the background expression in the non-stimulated cells from the BVDV stimulated cells. The increase in frequency of CD25^+^ labeling were determined by calculating the percent change by using the formula [(D-B)/B X 100], where B is the average frequency of CD25^+^ PBMCs for each respective viral strain in vaccinated calves and D is the average frequency of CD25^+^ PBMCs for each respective viral strain in non-vaccinated calves. The VNT were reported as log(2) transformed values. Figures were generated in Microsoft^®^ Excel for variables of interest and compared between vaccinated and non-vaccinated animals. The standard error of the mean was calculated using the standard error function in Microsoft^®^ Excel.

## Results

A higher frequency of CD25^+^ (IL-2α receptor) labeling was observed on PBMCs (Table 2) for vaccinated calves in both Groups 1 and 2 and for all BVDV strains used for stimulation, and a lower frequency was observed for non-vaccinated calves. For vaccinated calves in Group 1, an average of 54, 42, and 39% higher frequency than non-vaccinated calves was observed for CD25^+^ PBMCs when stimulated with BVDV-1a, BVDV-1b, and BVDV-2 strains, respectively (Table 2). For vaccinated calves in Group 2, a 42%, 14%, and 53% higher frequency than non-vaccinated calves was observed for CD25^+^ PBMCs when stimulated with BVDV-1a (PI407), BVDV-1b (Nebraska), and BVDV-2 (PI28) strains, respectively (Table 2). Similar IL-2 mRNA expression cell percentages were observed in the mitogen stimulated cells for both vaccinated and non-vaccinated calves (after 2-h stimulation) suggestive that the PBMC's were responsive to a stimulant (data not shown). Although, 24 h after stimulation with BVDV, regardless of vaccination status or BVDV strain used for stimulation, minimal IL-2 mRNA expression was observed. The lack of IL-2 expression in response to 24-h BVDV stimulation was observed in all specific PBMC subsets (data not shown). While an increase in CD25 labeling was observed in the vaccinated calves, the lack of IL-2 mRNA expression could be due to the timing of stimulation and analysis of the samples, suggesting 24 h post-BVDV stimulation may not be the optimal time to detect IL-2 mRNA expression.

**Table 2:** Average total frequency of CD25+ PBMCs for vaccinated and un-vaccinated calves in Groups 1 and 2 after stimulation with BVDV 1a, 1b, and 2a strains.

	**Virus**	**Non-vaccinates**	**Vaccinates**
**Group 1 calves**			
BVDV-1a	BAOEC1190	13.4	23
	GL760	17.2	23.9
	PI34	17.8	24.7
	PI407	13.4	23.9
BVDV-1b	Nebraska	15.2	23.1
	PI11	15.8	22.6
	PI285	16.3	21.5
	PI819	15.3	21.9
BVDV-2a	890	26.7	28.4
	AzSpleen	13.8	22.9
	PI28	14.8	23
	USMARC 60780	16.2	24.4
**Group 2 calves**			
BVDV-1a	PI407	10.3	14.8
BVDV-1b	Nebraska	9.8	11.3
BVDV-2a	PI28	9.2	14.2

**Figure 1:**
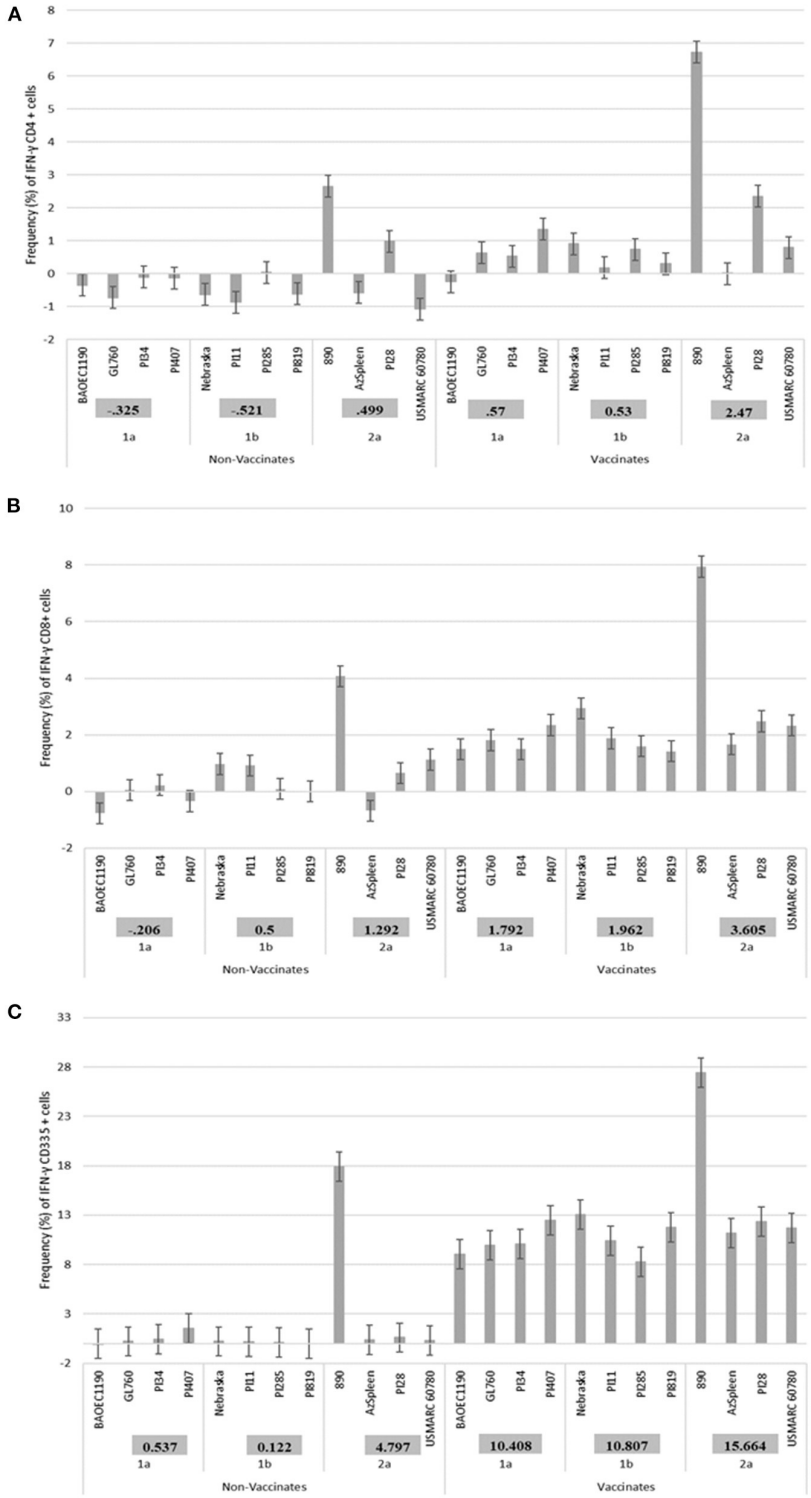
Percent positive cells for each respective PBMC subset **(A)** CD4^+^, **(B)** CD8^+^, **(C)** CD335^+^ for IFN-γ mRNA expression in Group 1 vaccinated and non-vaccinated calves at ~10–11 months post-initial vaccination and stimulated with 12 different BVDV strains (4 BVDV-1a, 4 BVDV-1b, and 4 BVDV-2a).

As previously reported, a higher IFN-γ mRNA expression in vaccinates and a lower expression was observed in non-vaccinated calves for the CD4^+^ and CD335^+^ PBMC subsets (27), therefore these two PBMC populations as well as the CD8^+^ subset were used for comparison in the current assessment. When 12 BVDV strains (4 BVDV-1a, 4 BVDV-1b and 4 BVDV-2a) were used for PBMCs isolated and stimulated from Group 1 calves, in general, higher IFN-γ mRNA expression in CD4^+^, CD8^+^, and CD335^+^ cells were observed in the vaccinated calves and lower expression the non-vaccinated calves (Figure 1). Additionally, in the vaccinated calves, variability in the level of expression of IFN-γ mRNA was observed for the respective 12 BVDV strains, suggesting a strain associated IFN-γ response in the CD4^+^ and CD8^+^ cells (Figures 1A,B) and to a lesser extent in the CD335^+^ cells (Figure 1C). In the vaccinated calves, the higher expression of IFN-γ mRNA was observed when stimulating with BVDV-1a (PI407), BVDV-1b (Nebraska), and BVDV-2a (890), and this was most pronounced in the CD4^+^ and CD8^+^ cells (Figures 1A,B) and less pronounced in the CD335^+^ cells (Figure 1C). The BVDV-2a strain 890 also induced expression of IFN-γ mRNA in the non-vaccinated calves. The expression of IFN-γ mRNA in the non-vaccinated calves may be due to the highly virulent nature of this strain and may be a non-specific response (Figure 1). The strains within each respective BVDV subgenotype, with the exclusion of 890 that was replaced with BVDV-2a strain PI28, were subsequently used in a second BVDV stimulation study. The 890 strain was not used in the second stimulation study given the IFN-γ mRNA expression in the non-vaccinated calves and the potential for a non-specific response associated with the virulence of this strain. Therefore, all strains used in the second stimulation study would be generally considered typical virulent strains.

**Figure 2:**
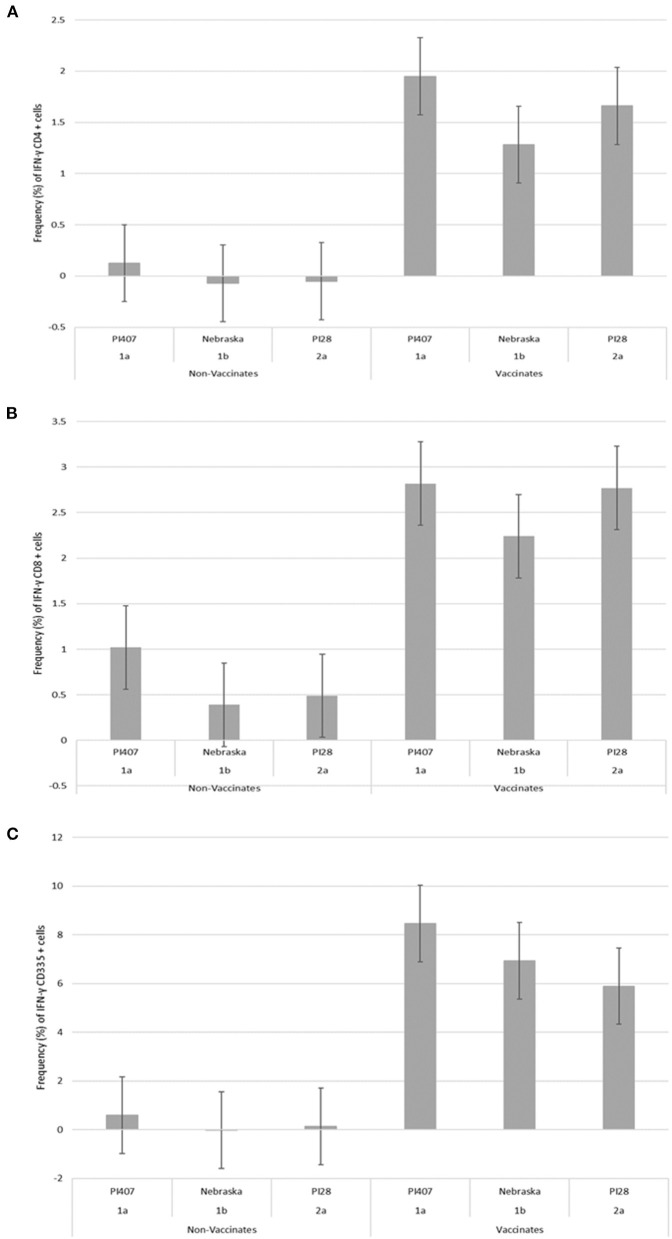
Percent positive cells for each respective PBMC subset **(A)** CD4^+^, **(B)** CD8^+^, **(C)** CD335^+^ for IFN-γ mRNA expression in Group 1 vaccinated and non-vaccinated calves at ~10–11 months post-initial vaccination and stimulated with three different BVDV strains (PI407 BVDV-1a, Nebraska BVDV-1b, and PI28 BVDV-2a).

**Figure 3:**
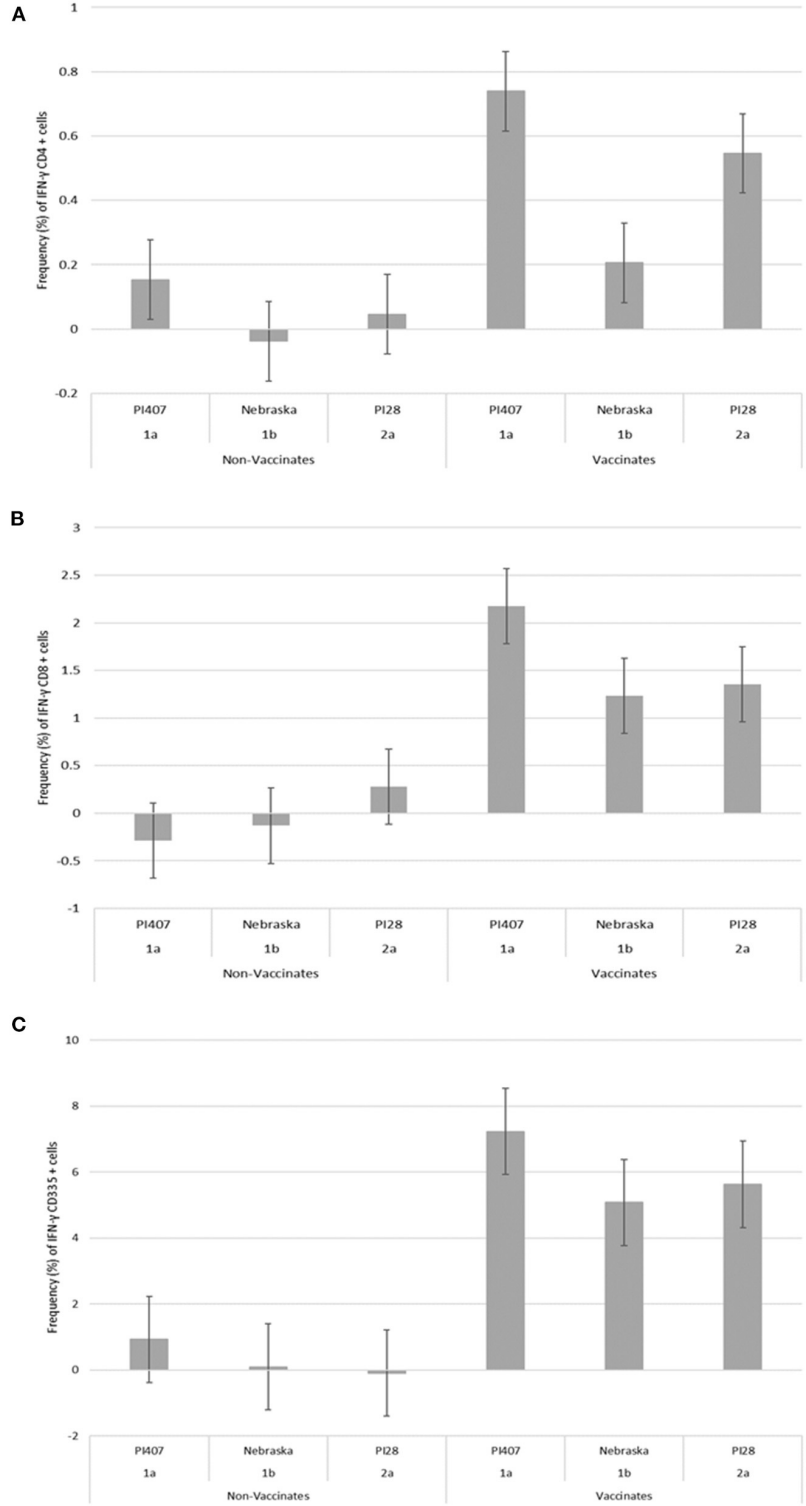
Percent positive cells for each respective PBMC subset **(A)** CD4^+^, **(B)** CD8^+^, **(C)** CD335^+^ for IFN-γ mRNA expression in Group 2 vaccinated and non-vaccinated calves at ~12 weeks post-initial vaccination and stimulated with three different BVDV strains (PI407 BVDV-1a, Nebraska BVDV-1b, and PI28 BVDV-2a).

PBMCs from calves in Group 1 were utilized in a subsequent stimulation study to corroborate results observed from the initial 12 virus stimulation study when using BVDV strains; BVDV-1a (PI407), BVDV-1b (Nebraska), and BVDV-2 (PI28). Additionally, PBMCs isolated from calves in Group 2 that had only received one dose of BVDV MLV vaccine were stimulated with the three strains BVDV-1a (PI407), BVDV-1b (Nebraska), and BVDV-2 (PI28). Higher IFN-γ mRNA expression in CD4^+^, CD8^+^, and CD335^+^ cells were observed in the vaccinated calves and lower expression in the non-vaccinated Group 1 calves (Figure 2). Similarly, the BVDV-1a and BVDV-2a strains induced higher IFN-γ mRNA expression in CD4^+^ and CD8^+^ cells and lower expression was observed for the BVDV-1b strain (Figures 2A,B). This trend in expression was not observed in the CD335^+^ cells (Figure 2C). Likewise, in the Group 2 calves, higher IFN-γ mRNA expression in CD4^+^, CD8^+^, and CD335^+^ cells were observed in the vaccinated calves and lower expression was observed in the non-vaccinated calves (Figure 3). In the Group 2 calves, the BVDV-1a and BVDV-2a strains induced higher IFN-γ mRNA expression and lower expression was observed for the BVDV-1b strain in all PBMC subsets evaluated (CD4^+^, CD8^+^, and CD335^+^ cells; Figure 3). While higher IFN-γ mRNA expression was observed in the vaccinated calves (Group 1 and 2), the lack of variability in addition to the lack of IFN-γ mRNA expression in the non-vaccinated calves suggests minimal background stimulation, indicating an antigen specific recall response to BVDV in the vaccinated calves for the PBMC subsets evaluated (Figures 1, 2, 3).

Serological responses as determined by VNT were also evaluated for calves in Groups 1 and 2. VNT were evaluated using serum from samples collected approximately 10-11 months after initial vaccination from Group 1 calves and approximately 12 weeks after vaccination for Group 2 calves. For calves in Group 1, serum samples were analyzed against all twelve BVDV strains and for calves in Group 2, serum samples were analyzed against the three BVDV strains BVDV-1a (PI407), BVDV-1b (Nebraska), and BVDV-2 (PI28) (Table 3). VNT differed from the CMI results in the Group 1 calves, but VNT and CMI results demonstrated similar trends in Group 2 calves. The average VNT for all BVDV-1a, BVDV-1b, and BVDV-2a viruses were 6.4, 6.6, and 9.9, respectively (Table 3). Regardless of all BVDV subgenotypes used in Group 1 calves, the lowest VNT was observed against the BVDV-1a PI407 strain and the highest VNT was observed against BVDV-2a strain 890 (Table 3). In Group 2 calves, the lowest VNT was against the BVDV-1b strain (Nebraska), and the highest VNT was against the BVDV-1a strain (PI407) (Table 3).

**Table 3:** Average virus neutralization titers (log2 transformed) from sera from vaccinated and non-vaccinated calves from Group 1 and 2 against BVDV 1a, 1b and 2a strains.

	**Virus**	**Non-vaccinates**	**Vaccinates**
**Group 1 calves**			
BVDV-1a	BAOEC1190	0	6.2
	GL760	0	7
	PI34	0	7
	PI407	0	5.4
BVDV-1b	Nebraska	0	7.5
	PI11	0	6.3
	PI285	0	6
	PI819	0	6.7
BVDV-2a	890	0	10.2
	AzSpleen	0	9.8
	PI28	0	9.7
	USMARC 60780	0	9.9
**Group 2 calves**			
BVDV-1a	PI407	0	5.7
BVDV-1b	Nebraska	0	2.7
BVDV-2a	PI28	0	3

## Discussion

Given the genetic diversity observed globally for BVDV, there is a recurring question regarding the association between genetic and antigenic relatedness. The basis of the question is rooted in the limited genetic and potentially antigenic diversity represented in currently available and licensed BVDV vaccines. While VNT titers reflect the existence of an immune response in response to an antigen, VNT may not be the definitive metric for determining efficacy and level of cross protection, As demonstrated in a paper examining cross protection against fetal infection between BVDV-1, BVDV-2, and a third bovine pestivirus, HoBi-like virus (36). In this study it was shown that pregnant dams, that had previously given birth to BVDV-1 or BVDV-2 persistently infected (PI) calves but were not PI themselves, had titers against HoBi-like virus that ranged from 1,448 to 5,793 at the time of exposure to HoBi-like virus. Despite these high titers, HoBi-like virus RNA was detected 30 days after inoculation in the challenged fetuses in all tested tissues. This indicated that despite a strong immune response against HoBi-like virus present in the dam, HoBi-like virus replicated and crossed the placenta to infect the fetus. These findings suggest that a BVDV vaccine, even one that would induce significantly more robust humoral immune response than those currently on the market, would likely fail to confer the necessary protection against HoBi-like viruses potentially due to the lack of antigenic similarity. While CMI was not measured in this study, it could be assumed that dams carrying a BVDV infected fetus would presumably mount a CMI response. Collectively, this would suggest that in order to confer fetal protection and effectively reduce viral replication, it is important that the immune response (both CMI and humoral) be targeted or specific to effectively reduce viral replication to prevent fetal infections.

As previously discussed, antigenic similarity is typically determined using serological assays such as VNT, whereas CMI is generally determined by the induction of IL-2, IFN-γ, and CD25 labeling (25–27, 29, 30). To date, no studies have measured differences in CMI responses against multiple BVDV strains to better understand if there is a strain specific component to CMI or if it is a generalized cellular response to BVDV antigens and contributes to a more broadly protective response. Data from the current study would suggest that the variability in CMI response is associated with the viral strain used for stimulation. Although, regardless of the BVDV subgenotype, variability in IFN-γ mRNA expression was most pronounced in the CD4^+^ population, this was observed between the viruses within each respective BVDV subgenotype in the Group 1 calves. Minimal to no detectable IFN-γ mRNA expression in the CD4^+^ population was observed for the BOAEC1190 (BVDV-1a) and AzSpleen (BVDV-2a) in the Group 1 calves. Whereas other BVDV-1a and BVDV-2a strains (PI407, PI28 and 890) induced the greatest IFN-γ mRNA expression in all the PBMC subsets evaluated (CD4^+^, CD8^+^, and CD335^+^) in the Group 1 calves. Although, these differences were not observed for frequency of CD25^+^ cells.

While, the frequency of CD25^+^ cells and IFN-γ mRNA expression in the CD8^+^ and CD335^+^ are not as notable between strains and this may be due to timing post stimulation, or these responses may be associated with a “general” BVDV CMI response which is less sensitive or obvious to differentiate among subgenotypes. This is most evident for the frequency of CD25^+^ cells, and the IFN-γ mRNA expression in the CD335^+^ population is less distinguishable between BVDV strains as compared to the other two cell populations (CD4^+^ and CD8^+^). Given the function of NK cells (CD335^+^) a more constant or magnitude of IFN-γ expression among the BVDV strains could be likely, as NK cells may not have an antigenic specific function but rather a more general response (37). Additionally, there may be an inverse relationship between VNT and IFN-γ mRNA expression, as the lowest VNT and highest IFN-γ mRNA expression was observed for the PI407 strain, whereas the highest VNT and lowest IFN-γ mRNA expression was observed for the BOAEC1190 strain, that may be strain and perhaps genotype specific.

Given that the current assay measures mRNA expression rather than protein accumulation over time for IL-2 and IFN-γ, potentially different BVDV strains may have a more rapid or delayed IFN-γ response and the lack of IL-2 response may be a direct result of timing post stimulation and detection of each respective cytokine, which is a limitation of the current assay. Other studies utilizing inactivated antigens for stimulation have reported peak IL-2 expression at ~ 8–16 h prior to IFN-γ expression (38). Since the current assay evaluated mRNA expression and not protein accumulation, the timing post stimulation is targeted at IFN-γ which is typically involved in CMI responses and has previously been optimized for the current assay (27). Although, this is a limiting factor of the current assay as multiple cytokines cannot be measured, but in general, IFN-γ is a characteristic cytokine produced by memory T cells during an antigen recall response (25–27, 29, 30, 39, 40). To this end, IFN-γ mRNA expression may vary in the different PBMCs evaluated in the current study, the timing may also vary in each respective PBMC population, and each BVDV strain may vary in timing of induction of IFN-γ mRNA expression. All these variables must be considered when evaluating the data from the current study. Given the potential variables that may impact IFN-γ mRNA expression, this lead to the rational to include two groups of vaccinated calves that vary in vaccination status, age, and breed. Additionally, this is the rational for initially evaluating 12 BVDV strains and choosing the three strains from each respective subgenotype that yielded the greatest IFN-γ mRNA expression in calves previously reported to have significant CMI responses as compared to non-vaccinated calves (27). Therefore, the results observed in each respective group of calves (Group 1 and 2) were collated to evaluate if similar trends were observed regardless of the frequency they were vaccinated, age, breed, or strain used for stimulation.

Group 1 calves that had received multiple doses of the respective MLV vaccines were initially used to make comparisons among the 12 BVDV strains. Subsequently, the BVDV strains within each BVDV subgenotype (BVDV-1,−1b, and−2a) were chosen to reevaluate Group 1 calves and evaluate Group 2 calves that only received one dose of MLV vaccine to determine if similar trends exist among calves that differ in the frequency they were vaccinated, age, and breed. A trend existed within the Group 1 calves that received multiple doses of BVDV vaccines for the BVDV-1b strain (Nebraska) to have lower IFN-γ mRNA expression, this trend was also observed in the Group 2 calves. In the Group 2 calves that only received one dose of BVDV vaccine, lower IFN-γ mRNA expression in the CD4^+^ population for the BVDV-1b strain was observed, and to a lesser extent in the CD8^+^ and CD335^+^ cell population and CD25 labeling. Interestingly, similar to the cell mediated data in the Group 1 calves that received multiple doses of the vaccine, potential trends for antigenic differences were less distinguishable among the BVDV-1 strains when evaluating VNT data. Although, similar antigenic trends existed for both humoral and cell mediated data with both the VNT and the IFN-γ mRNA expression being lower for the BVDV-1b strain in the Group 2 calves. Collectively, this data would support the existence of differences in how the immune system responds to each respective BVDV strain, but also indicates with regard to CMI, there may be a targeted antigenic response rather than just a “general” CMI response. Furthermore, a better understanding of BVDV strains used for evaluating cell mediated responses in needed given the variability that was observed in the current study. It is unknown what level of cell mediated response is needed to provide a protective immune response. While the IFN-γ mRNA expression differed among BVDV strains, in general, vaccinated calves tended to have higher IFN-γ expression and lower IFN-γ expression was observed in non-vaccinated calves. As with VNT and the titer needed to confer protection, it is unknown if there is a level of CMI that is needed, or if any measurable CMI response is adequate for contributing to conferring fetal protection for BVDV. Additionally, the IFN-γ mRNA expression in the CD4^+^, CD8^+^, and CD335^+^ cell populations in vaccinated calves may vary in each respective PBMC population, but all populations are contributing to the collective cell mediated response and perhaps protective responses. Therefore, the differences in IFN-γ mRNA expression in each cell population may not have implications on protection but these differences may be due to epitope repertoire frequency and recognition after vaccination and subsequent stimulation. Previous reports have demonstrated that CD8 T cell responses target mainly NS3 protein, followed by Capsid, NS5 and NS4A/B proteins for Dengue infection (39). Conversely, CD4 T cell responses target mainly Capsid, followed by Envelope, NS3, NS2A/B, and NS5 proteins for Dengue infection (39). Additionally, protein immunodominance for both CD4 and CD8 T cells in Dengue virus infection is also a function of multiple exposure of Dengue infection, and that tends to skew protein immunodominance toward epitopes highly conserved across different Dengue serotypes (41). Collectively, this would suggest T cell protein/epitope immunodominance is complex and widely focuses on multiple protein targets/epitopes. Therefore, to mount and efficient antigen specific T cell responses multiple proteins/epitopes are necessary for an effective response, although it may be that immunodominant epitopes may not completely correlate to protective epitopes.

Previous reports have suggested that BVDV E2, NS2-3, and the N-terminal protease fragment of the N^pro^ proteins contain CD4^+^ T cell epitopes, and MHC class II DR-restricted T cell epitopes have been identified from conserved regions of E2 and NS2-3 (42). While T cell epitopes have been suggested for BVDV, the identification of all potential BVDV T cell epitopes are unknown. A collection of 573 overlapping peptides spanning 82% of the amino acid sequence of classical swine fever virus (CSFV) identified 26 peptide sequences containing T cell epitopes (43). The T cell epitopes identified for CSFV spanned across multiple pestivirus proteins including; E^rns^, E1, E2, NS2-3, NS4A, NS4B, NS5A (43). Therefore, it would be hypothesized that T cell epitopes would also span multiple BVDV proteins rather than just E2, NS2-3 and N^pro^. While multiple T cell epitopes may span the BVDV genome, it is unknown if there are immunodominant epitopes or if there are BVDV species or subgenotype dominant epitopes as observed with Dengue (39, 41). Data from the current study suggest there may be immunodominant epitopes or antigen specific T cell responses, as cattle that received MLV vaccines that contain BVDV 1a and 2a antigens have greater IFN-γ mRNA expression to these two antigens post stimulation and lower IFN-γ mRNA expression to the BVDV-1b strain not contained in the MLV vaccines. Protective immune response may include both “general” and “targeted species/genotype/strain” specific antibodies and CMI responses. The immunological pressure to induce both a general and a targeted response would be to eliminate the infecting strain as effectively as possible and also provide protection against infection with related strains or mutation occurring in the original infecting virus strain. High specific immune responses may equate with efficient clearing of the initial infecting virus but being too specific would make the immune response less efficient at clearing other BVDV strains. It is unknown if this type of immune response could be due to variability in immune response among animals, or if this is associated with specific BVDV strains that interact with the immune system differently?

More data is required to better understand the collective immune responses, both humoral and cell mediated, as it relates to protection, antigen specific responses, and potential recommendations for vaccination practices to contribute to control of BVDV. More work is needed to better understand the consequences of inducing specific or general humoral and cell mediated responses and the implications as it relates to conferring protection against antigenically diverse BVDV strains. Furthermore, more research is needed to understand the differences in CMI responses induced by various MLV and inactivated BVDV vaccines for recommendations related use of vaccines in control programs. It is unknown if similar responses would be observed given the strain differences and adjuvants used in each respective licensed BVDV vaccine.

## Data Availability Statement

The raw data supporting the conclusions of this article will be made available by the authors, without undue reservation.

## Ethics Statement

The animal study was reviewed and approved by Institutional Animal Care and Use Committee of the National Animal Disease Center. Written informed consent was obtained from the owners for the participation of their animals in this study.

## Author Contributions

SF, RD, BT, JFR, and JAR: conceived and designed the experiment. SF, RD, and BT: performed the experiment. SF, RD, JAR, and JFR: analyzed the data. JN and BT: contributed reagents, materials, and analysis tools. SF: wrote the paper. RD, BT, JFR, JN, and JAR: reviewed the paper. All authors contributed to the article and approved the submitted version.

## Conflict of Interest

JFR was employed by company Ridpath Consulting, LLC. The remaining authors declare that the research was conducted in the absence of any commercial or financial relationships that could be construed as a potential conflict of interest.

## References

[1] Evans CA Pinior B Larska M Graham D Schweizer M Guidarini C. Global knowledge gaps in the prevention and control of bovine viral diarrhoea (BVD) virus. Transbound Emerg Dis. (2019) 66:640–52. 10.1111/tbed.13068 30415496

[2] Scharnböck B Roch F-F Richter V Funke C Firth CL Obritzhauser W. A meta-analysis of bovine viral diarrhoea virus (BVDV) prevalences in the global cattle population. Sci Rep. (2018) 8:14420. 10.1038/s41598-018-32831-2 30258185 PMC6158279

[3] Ridpath JF. Immunology of BVDV vaccines. Biologicals. (2013) 41:14–9. 10.1016/j.biologicals.2012.07.003 22883306

[4] Lindberg A Houe H. Characteristics in the epidemiology of bovine viral diarrhea virus (BVDV) of relevance to control. Prev Vet Med. (2005) 72:55–73. 10.1016/j.prevetmed.2005.07.018 16214248

[5] Deregt D. Introduction and history. In: Ridpath J Goyal S, editors. Bovine Viral Diarrhea Virus: Diagnosis, Management, and Control. Hoboken, NJ: Blackwell Publishing (2005). p. 3–33.

[6] Houe H Lindberg A Moennig V. Test strategies in bovine viral diarrhea virus control and eradication campaigns in Europe. J Vet Diagn Investig. (2006) 18:427–36. 10.1177/104063870601800501 17037609

[7] Smith DB Meyers G Bukh J Gould EA Monath T Muerhoff AS. Proposed revision to the taxonomy of the genus Pestivirus, family Flaviviridae. J Gen Virol. (2017) 98:2106. 10.1099/jgv.0.000873 28786787 PMC5656787

[8] Yeşilbag K Alpay G Becher P. Variability and global distribution of subgenotypes of bovine viral diarrhea virus. Viruses. (2017) 9:128. 10.3390/v9060128 28587150 PMC5490805

[9] Ridpath J Bolin S Dubovi E. Segregation of bovine viral diarrhea virus into genotypes. Virology. (1994) 205:66–74. 10.1006/viro.1994.1620 7975238

[10] Brock K Cortese V. Experimental fetal challenge using type II bovine viral diarrhea virus in cattle vaccinated with modified-live virus vaccine. Vet Ther. (2001) 2:354–60. 19746658

[11] Fulton RW Whitley EM Johnson BJ Ridpath JF Kapil S Burge LJ. Prevalence of bovine viral diarrhea virus (BVDV) in persistently infected cattle and BVDV subtypes in affected cattle in beef herds in south central United States. Can J Vet Res. (2009) 73:283. 20046630 PMC2757709

[12] Workman AM Heaton MP Harhay GP Smith TP Grotelueschen DM Sjeklocha D. Resolving Bovine viral diarrhea virus subtypes from persistently infected US beef calves with complete genome sequence. J Vet Diagn Investig. (2016) 28:519–28. 10.1177/1040638716654943 27400958

[13] Neill JD Crossley BM Mosena AC Ridpath JF Bayles DO Hietala SK. Genomic and antigenic characterization of a cytopathic bovine viral diarrhea virus 1i isolated in the United States. Virology. (2019) 535:279-82. 10.1016/j.virol.2019.07.020 31357167

[14] Neill JD Workman AM Hesse R Bai J Porter EP Meadors B. Identification of BVDV2b and 2c subgenotypes in the United States: genetic and antigenic characterization. Virology. (2019) 528:19-29. 10.1016/j.virol.2018.12.002 30553108

[15] Couvreur B Letellier C Collard A Quenon P Dehan P Hamers C. Genetic and antigenic variability in bovine viral diarrhea virus (BVDV) isolates from Belgium. Virus Res. (2002) 85:17–28. 10.1016/S0168-1702(02)00014-X 11955635

[16] Bachofen C Stalder H Braun U Hilbe M Ehrensperger F Peterhans E. Co-existence of genetically and antigenically diverse bovine viral diarrhoea viruses in an endemic situation. Vet Microbiol. (2008) 131:93–102. 10.1016/j.vetmic.2008.02.023 18424020

[17] Minami F Nagai M Ito M Matsuda T Takai H Jinkawa Y. Reactivity and prevalence of neutralizing antibodies against Japanese strains of bovine viral diarrhea virus subgenotypes. Comp Immunol Microbiol Infect Dis. (2011) 34:35–9. 10.1016/j.cimid.2009.10.007 19931181

[18] Ridpath JF Fulton RW Kirkland PD Neill JD. Prevalence and antigenic differences observed between Bovine viral diarrhea virus subgenotypes isolated from cattle in Australia and feedlots in the southwestern United States. J Vet Diagn Invest. (2010) 22:184–91. 10.1177/104063871002200203 20224075

[19] Becher P Ramirez RA Orlich M Rosales SC König M Schweizer M. Genetic and antigenic characterization of novel pestivirus genotypes: implications for classification. Virology. (2003) 311:96–104. 10.1016/S0042-6822(03)00192-2 12832207

[20] Mosena ACS Falkenberg SM Ma H Casas E Dassanayake RP Walz PH. Multivariate analysis as a method to evaluate antigenic relationships between BVDV vaccine and field strains. Vaccine. (2020) 38:5764–72. 10.1016/j.vaccine.2020.07.010 32690424

[21] Beer M Hehnen H-R Wolfmeyer A Poll G Kaaden O-R Wolf G. A new inactivated BVDV genotype I and II vaccine: an immunisation and challenge study with BVDV genotype I. Vet Microbiol. (2000) 77:195–208. 10.1016/S0378-1135(00)00276-5 11042413

[22] Bolin S Ridpath J. Assessment of protection from systemic infection or disease afforded by low to intermediate titers of passively acquired neutralizing antibody against bovine viral diarrhea virus in calves. Am J Vet Res. (1995) 56:755–9. 7653884

[23] Walz PH Givens MD Rodning SP Riddell KP Brodersen BW Scruggs D. Evaluation of reproductive protection against bovine viral diarrhea virus and bovine herpesvirus-1 afforded by annual revaccination with modified-live viral or combination modified-live/killed viral vaccines after primary vaccination with modified-live viral vaccine. Vaccine. (2017) 35:1046–54. 10.1016/j.vaccine.2017.01.006 28111144

[24] Rodning SP Marley MSD Zhang Y Eason AB Nunley CL Walz PH. Comparison of three commercial vaccines for preventing persistent infection with bovine viral diarrhea virus. Theriogenology. (2010) 73:1154–63. 10.1016/j.theriogenology.2010.01.017 20181385

[25] Platt R Burdett W Roth JA. Induction of antigen-specific T-cell subset activation to bovine respiratory disease viruses by a modified-live virus vaccine. Am J Vet Res. (2006) 67:1179–84. 10.2460/ajvr.67.7.1179 16817740

[26] Platt R Widel PW Kesl LD Roth JA. Comparison of humoral and cellular immune responses to a pentavalent modified live virus vaccine in three age groups of calves with maternal antibodies, before and after BVDV type 2 challenge. Vaccine. (2009) 27:4508–19. 10.1016/j.vaccine.2009.05.012 19446589

[27] Falkenberg SM Dassanayake RP Neill JD Walz PH Casas E Ridpath JF. Measuring CMI responses using the PrimeFlow RNA assay: a new method of evaluating BVDV vaccination response in cattle. Vet Immunol Immunopathol. (2020) 221:110024. 10.1016/j.vetimm.2020.110024 32070831

[28] Walz PH Riddell KP Newcomer BW Neill JD Falkenberg SM Cortese VS. Comparison of reproductive protection against bovine viral diarrhea virus provided by multivalent viral vaccines containing inactivated fractions of bovine viral diarrhea virus 1 and 2. Vaccine. (2018) 36:3853–60. 10.1016/j.vaccine.2018.04.005 29699786

[29] Stevens E Zimmerman A Butterbaugh R Barling K Scholz D Rhoades J. The induction of a cell-mediated immune response to bovine viral diarrhea virus with an adjuvanted inactivated vaccine. Vet Ther. (2009) 10:E1–8. 20425730

[30] Van Anne TR Rinehart CL Buterbaugh RE Bauer MJ Young AJ Blaha ML. Cell-mediated and humoral immune responses to bovine herpesvirus type 1 and bovine viral diarrhea virus in calves following administration of a killed-virus vaccine and bovine herpesvirus type 1 challenge. Am J Vet Res. (2018) 79:1166–78. 10.2460/ajvr.79.11.1166 30372148

[31] Bauermann FV Flores EF Falkenberg SM Weiblen R Ridpath JF. Lack of evidence for the presence of emerging HoBi-like viruses in North American fetal bovine serum lots. J Vet Diagn Investig. (2014) 26:10–7. 10.1177/1040638713518208 24415196

[32] Bauermann FV Flores EF Ridpath JF. Antigenic relationships between Bovine viral diarrhea virus 1 and 2 and HoBi virus. J Vet Diagn Invest. (2012) 24:253–61. 10.1177/1040638711435144 22379042

[33] Bauermann FV Ridpath JF Weiblen R Flores EF. HoBi-like viruses: an emerging group of pestiviruses. J Vet Diagn Invest. (2013) 25:6–15. 10.1177/1040638712473103 23345268

[34] Fulton RW Confer A Burge LJ Perino LJ d'Offay J Payton ME. Antibody responses by cattle after vaccination with commercial viral vaccines containing bovine herpesvirus-1, bovine viral diarrhea virus, parainfluenza-3 virus, and bovine respiratory syncytial virus immunogens and subsequent revaccination at day 140. Vaccine. (1995) 13:725–33. 10.1016/0264-410X(94)00072-U 7483787

[35] Ridpath J Neill J Frey M Landgraf J. Phylogenetic, antigenic and clinical characterization of type 2 BVDV from North America. Vet Microbiol. (2000) 77:145–55. 10.1016/S0378-1135(00)00271-6 11042408

[36] Bauermann F Falkenberg S Ridpath J. HoBi-like virus RNA detected in foetuses following challenge of pregnant cows that had previously given birth to calves persistently infected with bovine viral diarrhoea virus. Transbound Emerg Dis. (2017) 64:1624–32. 10.1111/tbed.12556 27615437

[37] Boysen P Olsen I Berg I Kulberg S Johansen GM Storset AK. Bovine CD2-/NKp46+ cells are fully functional natural killer cells with a high activation status. BMC Immunol. (2006) 7:1–10. 10.1186/1471-2172-7-10 16643649 PMC1482717

[38] Harrington NP Surujballi OP Prescott JF. Cervine (Cervus elaphus) cytokine mRNA quantification by real-time polymerase chain reaction. J Wildlife Dis. (2006) 42:219–33. 10.7589/0090-3558-42.2.219 16870845

[39] Tian Y Grifoni A Sette A Weiskopf D. Human T cell response to dengue virus infection. Front Immunol. (2019) 10:2125. 10.3389/fimmu.2019.02125 31552052 PMC6737489

[40] Swain SL McKinstry KK Strutt TM. Expanding roles for CD4+ T cells in immunity to viruses. Nat Rev Immunol. (2012) 12:136–48. 10.1038/nri3152 22266691 PMC3764486

[41] Weiskopf D Angelo MA Sidney J Peters B Shresta S Sette A. Immunodominance changes as a function of the infecting dengue virus serotype and primary versus secondary infection. J Virol. (2014) 88:11383–94. 10.1128/JVI.01108-14 25056881 PMC4178794

[42] Lokhandwala S Fang X Waghela SD Bray J Njongmeta LM Herring A. Priming cross-protective bovine viral diarrhea virus-specific immunity using live-vectored mosaic antigens. PLoS ONE. (2017) 12:e0170425. 10.1371/journal.pone.0170425 28099492 PMC5242483

[43] Armengol E Wiesmüller K-H Wienhold D Büttner M Pfaff E Jung G. Identification of T-cell epitopes in the structural and non-structural proteins of classical swine fever virus. J Gen Virol. (2002) 83:551–60. 10.1099/0022-1317-83-3-551 11842250

